# Supplementary figures and images for: Desert-adapted tomato Solanum pennellii exhibit unique regulatory elements and stress-ready transcriptome patterns to drought
Source: PLoS One. 2025 May 20;20(5):e0324724. doi: 10.1371/journal.pone.0324724 (PMC12091751; doi:10.1371/journal.pone.0324724)

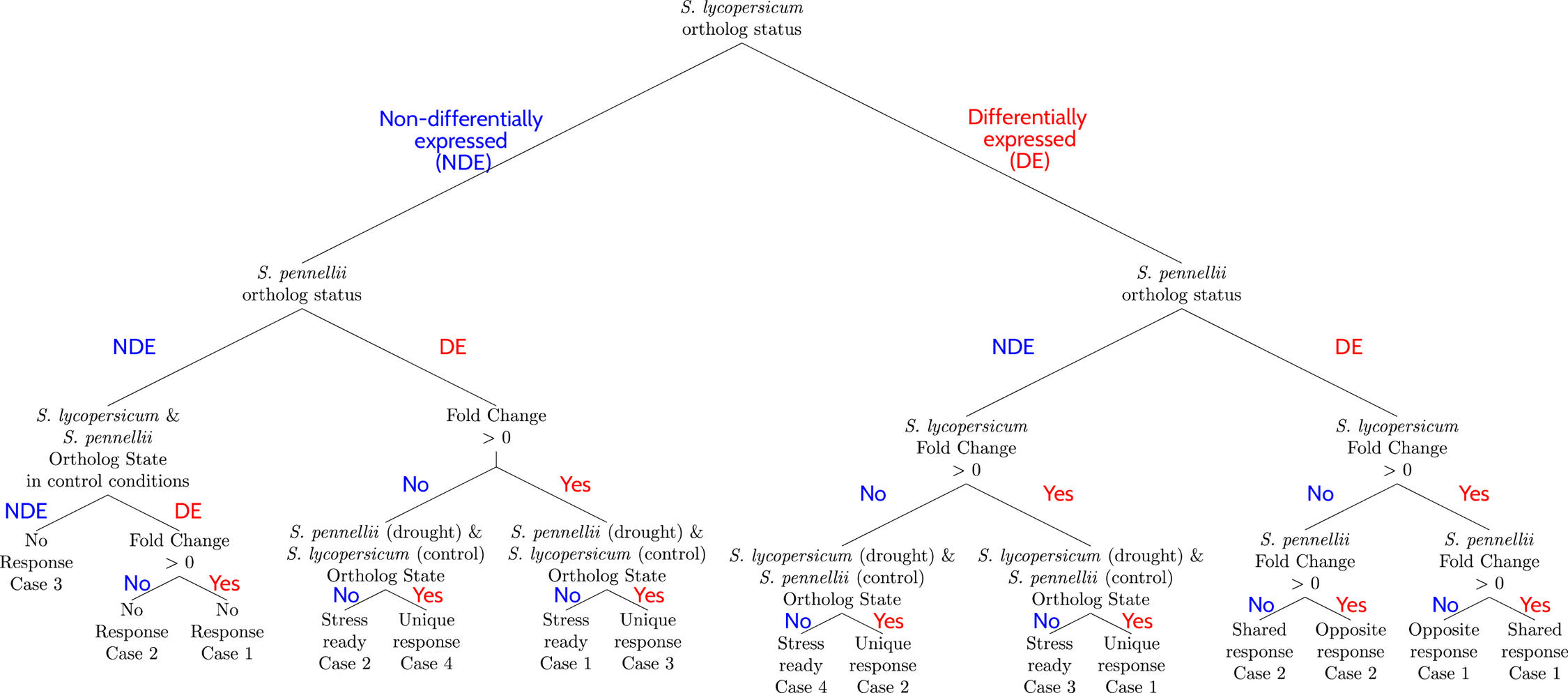

Supplement: S1 Fig — The diagram shows a series of binary decisions based on intra- and inter-species gene expression criteria and fold change to categorize ortholog genes into specific expression models. (TIF) [file pone.0324724.s001.tif]

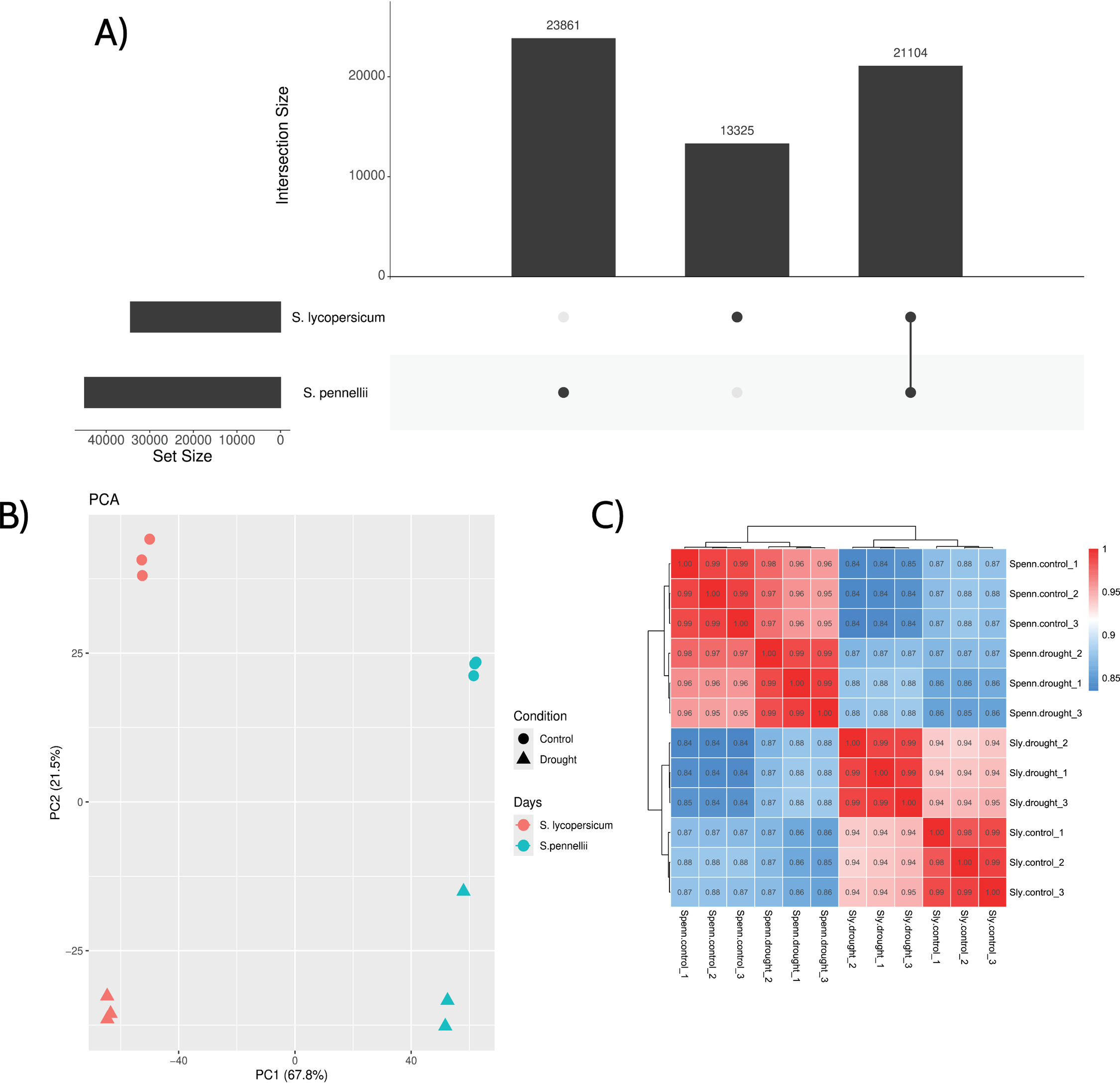

Supplement: S2 Fig — A) Number of orthologs and genes with and without an ortholog. B) Principal Component Analysis of orthologs expression under control and drought conditions, showing a clear separation between specie (Principal Component 1) and condition (Principal Component 2). C) of orthologs expression under control and drought conditions. (TIF) [file pone.0324724.s002.tif]

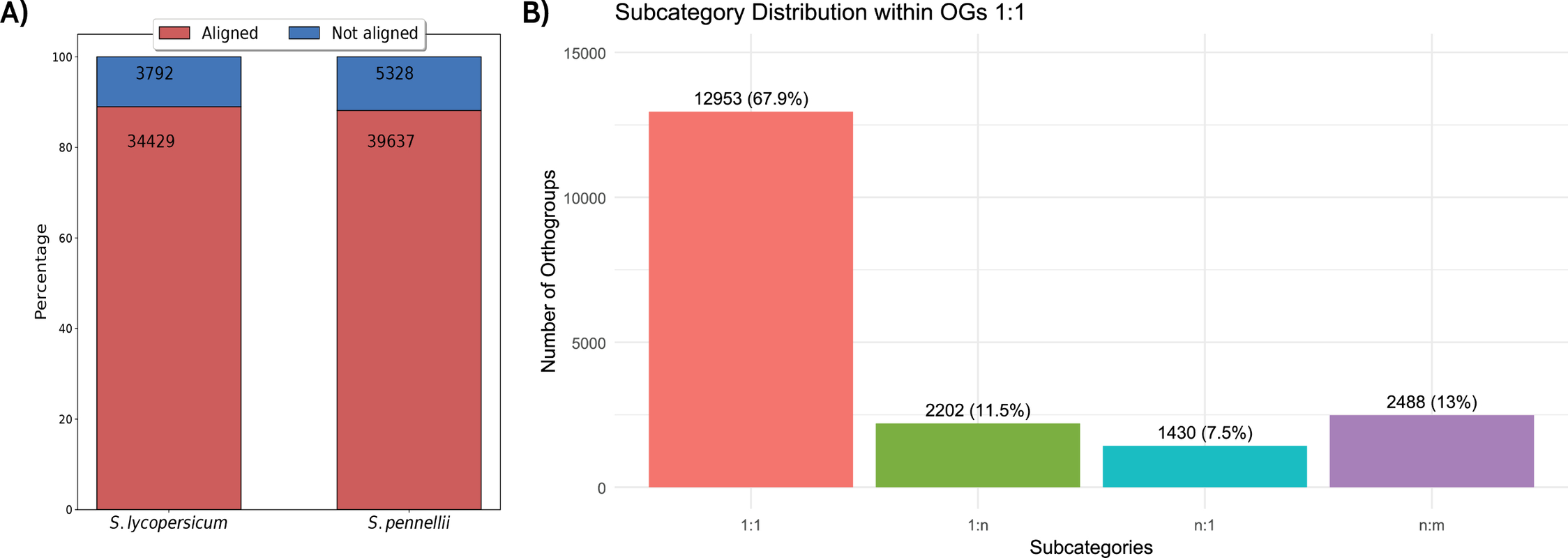

Supplement: S3 Fig — A) Graph shows the Number and percentage of genes with and without alignment in the other species (red and blue bar respectively). The results highlight a high proportion of genes in each species with corresponding homologous genes in the other species. B) Classification of orthologous gene relationships given by Orthofinder, depicting the distribution of one-to-one, one-to-many, and many-to-many orthologous gene mappings between S. lycopersicum and S. pennellii. (TIF) [file pone.0324724.s003.tif]

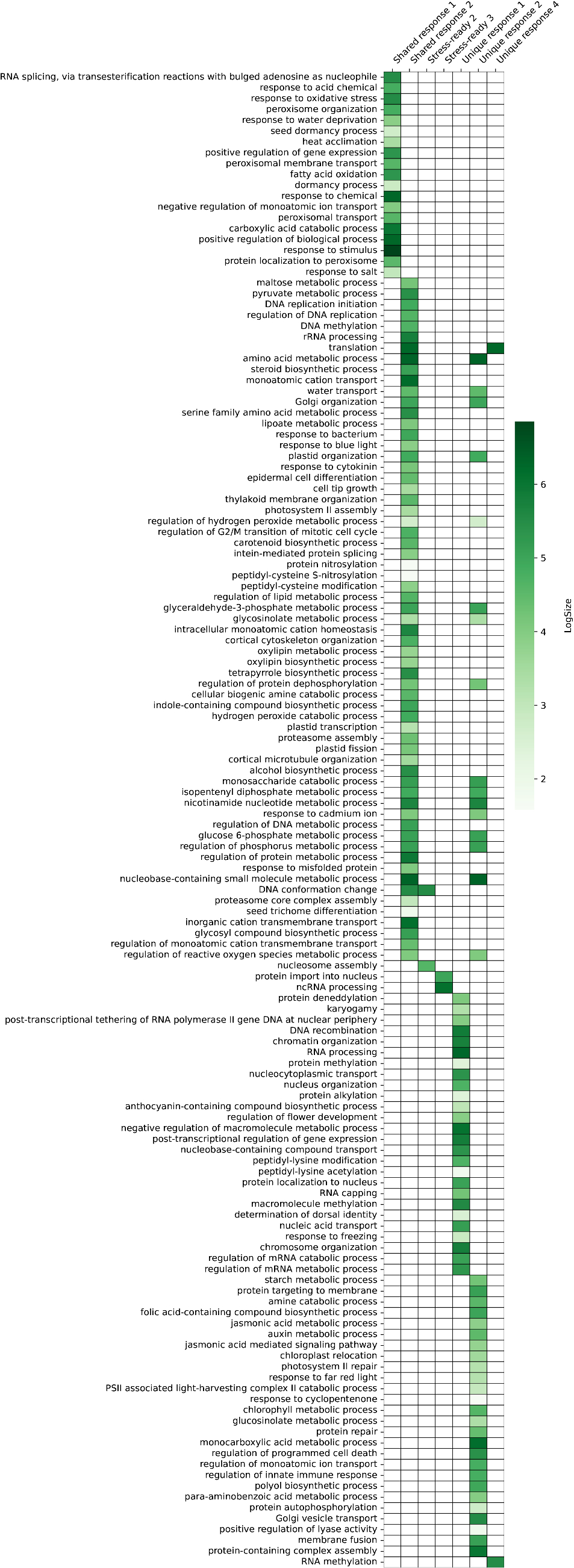

Supplement: S4 Fig — The heatmap highlights that most biological processes (rows) are distinct, with partial overlap observed in RNA metabolic processes and energy functions between responses. Green shades indicate −log10(adjusted P-value). (TIF) [file pone.0324724.s004.tif]

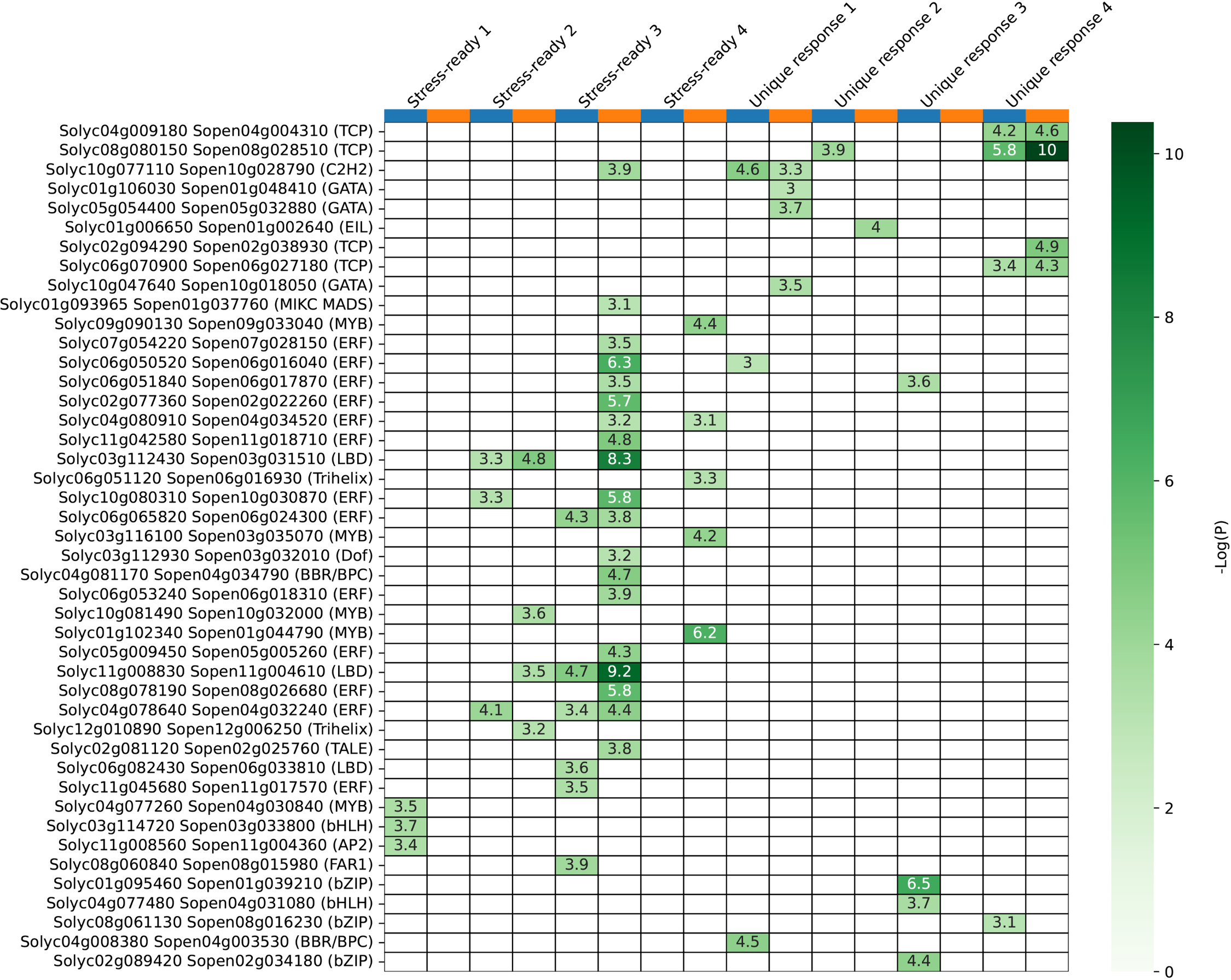

Supplement: S5 Fig — Rows represent TF ortholog in both S. lycopersicum and S. pennellii, along with their respective TF families, while columns correspond to the different stress-ready and unique response cases in S. lycopersicum (blue rectangles) and S. pennellii (orange rectangles). Green heatmap indicates p-values on a -log scale. (TIF) [file pone.0324724.s005.tif]

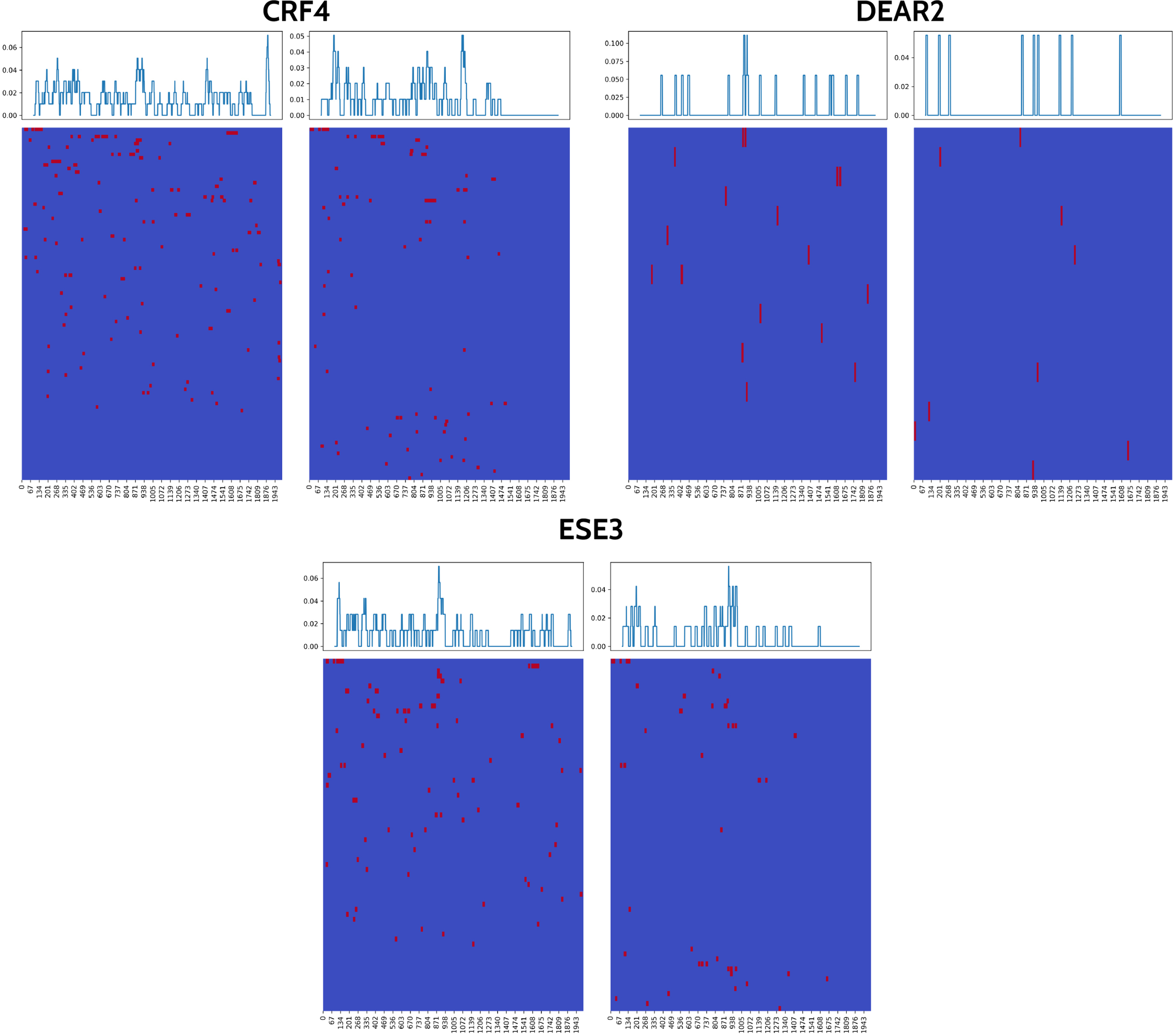

Supplement: S6 Fig — The upper panel shows the frequency of occurrence in the promoters, while the lower panel shows the position for each ortholog. (TIF) [file pone.0324724.s006.tif]

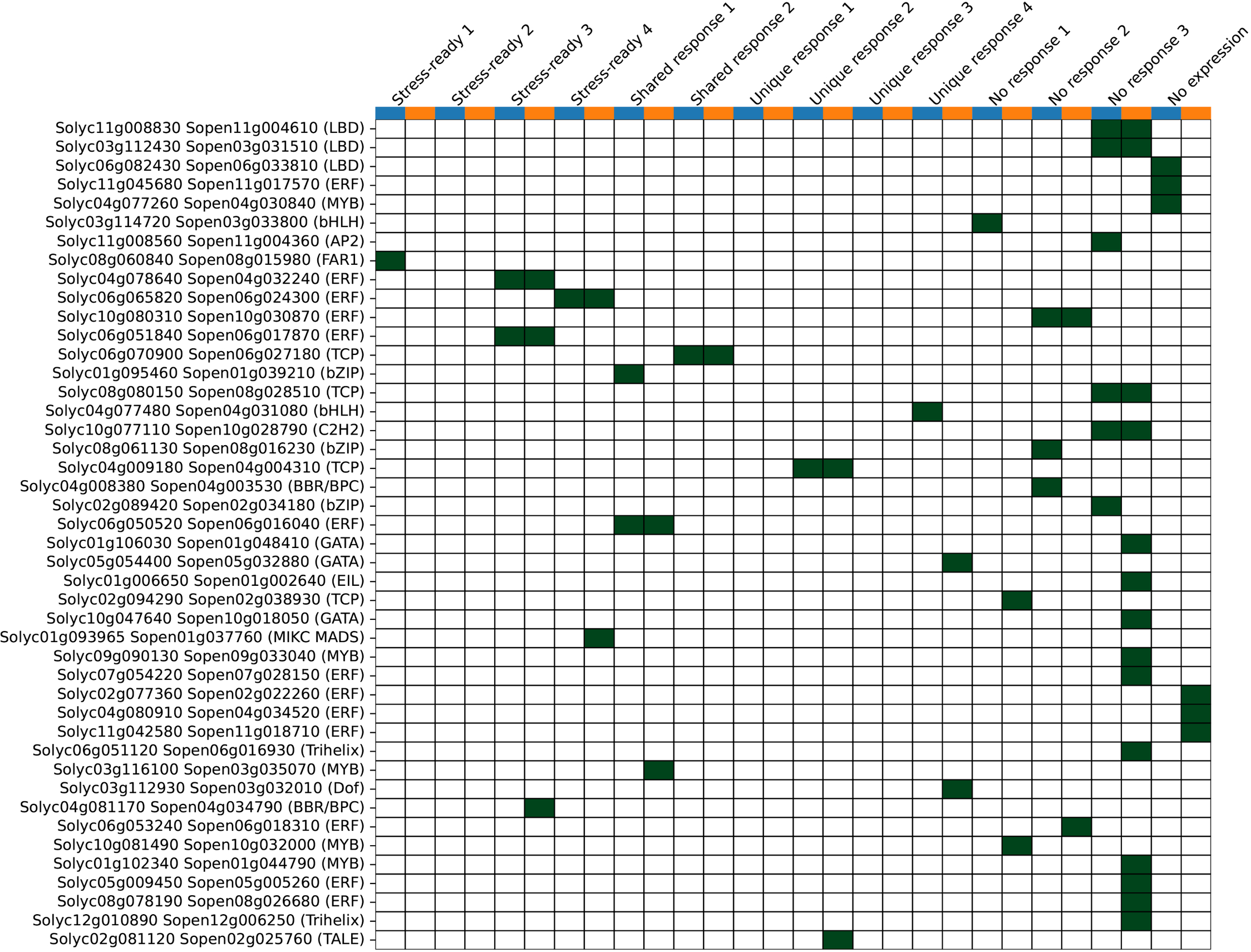

Supplement: S7 Fig — Rows represent TFBMs enriched in promoters, as shown in S5 Fig. Columns correspond to the different stress-ready and unique response cases in S. lycopersicum (blue rectangles) or S. pennellii (orange rectangles). Green shades indicate the group affiliation of the current TF. (TIF) [file pone.0324724.s007.tif]
