# Supplementary material for: Desert-adapted tomato Solanum pennellii exhibit unique regulatory elements and stress-ready transcriptome patterns to drought
Source: PLoS One. 2025 May 20;20(5):e0324724. doi: 10.1371/journal.pone.0324724 (PMC12091751; doi:10.1371/journal.pone.0324724)
Supplement: Attachment — Submitted filename: Response to Reviewers.docx [file pone.0324724.s017.docx]

Mohammad Irfan, Ph.D.

Academic Editor

PLOS ONE

Dear Editor

We sincerely thank the editor for the opportunity to resubmit and the reviewers for their valuable comments and suggestions regarding our manuscript (ID: PONE-D-25-00360, titled “Desert-adapted tomato *Solanum pennellii* exhibit unique regulatory elements and stress-ready transcriptome patterns to drought”). We have thoroughly addressed each point raised and revised the manuscript accordingly and, in addition, we believe that the revisions have significantly enhanced the clarity and overall quality of the manuscript.

Additionally, as suggested, we would like to modify our financial disclosure as follows

"This work was funded by grants FONDECYT-1210389 and 1250403 (to JMA), FONDECYT-3220673 (to JSCR), FONDECYT-3220801 (to TM), ANID Millennium Nucleus in Data Science and Plant Resilience (PhytoLearning) NCN2024_047, and Instituto Milenio de Biología Integrativa iBio Chile ICN17_002 from the Agencia Nacional de Investigación y Desarrollo de Chile (ANID). JMA's lab is also supported by the National Science Foundation (NSF) Plant Genome Grant NSF-PGRP: IOS-1840761. JMJG is funded by the Ministerio de Ciencia, Innovación y Universidades of Spain (PID2023-151867OB-C33) and the European Union's HORIZON-EIC-PATHFINDEROPEN no. 101098680 (project DARkWIN). This research was partially supported by the supercomputing infrastructure of the NLHPC (ECM-02). The funders had no role in study design, data collection and analysis, decision to publish, or preparation of the manuscript."

Below, we provide detailed responses to the reviewers' comments (highlighted in blue). Please note that all line and page references in our responses correspond to the revised version of the manuscript without tracked changes.

======================================================================

PONE-D-25-00360

Desert-adapted tomato Solanum pennellii exhibit unique regulatory elements and stress-ready transcriptome patterns to drought.

PLOS ONE

Dear Dr. Alvarez,

Thank you for submitting your manuscript to PLOS ONE. After careful consideration, we feel that it has merit but does not fully meet PLOS ONE’s publication criteria as it currently stands. Therefore, we invite you to submit a revised version of the manuscript that addresses the points raised during the review process.

The manuscript was reviewed by two independent experts in the field. Both the reviewers found the work interesting but raised several issues, which should be addressed for further consideration. The reviewers provide detailed comments in their reviews and point out the areas where the manuscript needs to be improved.

Please submit your revised manuscript by Apr 14 2025 11:59PM. If you will need more time than this to complete your revisions, please reply to this message or contact the journal office at plosone@plos.org. When you're ready to submit your revision, log on to https://www.editorialmanager.com/pone/ and select the 'Submissions Needing Revision' folder to locate your manuscript file.

Please include the following items when submitting your revised manuscript:

A rebuttal letter that responds to each point raised by the academic editor and reviewer(s). You should upload this letter as a separate file labeled 'Response to Reviewers'.

We have included it.

A marked-up copy of your manuscript that highlights changes made to the original version. You should upload this as a separate file labeled 'Revised Manuscript with Track Changes'.

We have included it.

An unmarked version of your revised paper without tracked changes. You should upload this as a separate file labeled 'Manuscript'.

We have included it.

If you would like to make changes to your financial disclosure, please include your updated statement in your cover letter. Guidelines for resubmitting your figure files are available below the reviewer comments at the end of this letter.

If applicable, we recommend that you deposit your laboratory protocols in protocols.io to enhance the reproducibility of your results. Protocols.io assigns your protocol its own identifier (DOI) so that it can be cited independently in the future. For instructions see: https://journals.plos.org/plosone/s/submission-guidelines#loc-laboratory-protocols. Additionally, PLOS ONE offers an option for publishing peer-reviewed Lab Protocol articles, which describe protocols hosted on protocols.io. Read more information on sharing protocols at https://plos.org/protocols?utm_medium=editorial-email&utm_source=authorletters&utm_campaign=protocols.

We look forward to receiving your revised manuscript.

Kind regards,

Mohammad Irfan, Ph.D.

Academic Editor

PLOS ONE

Journal Requirements:

When submitting your revision, we need you to address these additional requirements.

1.Please ensure that your manuscript meets PLOS ONE's style requirements, including those for file naming. The PLOS ONE style templates can be found at

https://journals.plos.org/plosone/s/file?id=wjVg/PLOSOne_formatting_sample_main_body.pdf and

https://journals.plos.org/plosone/s/file?id=ba62/PLOSOne_formatting_sample_title_authors_affiliations.pdf

2. Thank you for stating the following financial disclosure:

“This work was funded by grants FONDECYT-1210389 and 1250403 (JMA) FONDECYT-3220673 (to JSCR), FONDECYT-3220801 (to TM), ANID Millennium Nucleus in Data Science and Plant Resilience (PhytoLearning) NCN2024_047, and Instituto Milenio de Biología Integrativa iBio Chile ICN17_002 from “Agencia Nacional de Investigación y Desarrollo de Chile (ANID)”. JMA lab is also supported by National Science Foundation (NSF) Plant Genome Grant NSF-PGRP: IOS-1840761. JMJG is funded by Ministerio de Ciencia Inovación y Universidades of Spain (PID2023-151867OB-C33) and European Union's HORIZON-EIC-PATHFINDEROPEN no. 101098680 (project DARkWIN). This research was partially supported by the supercomputing infrastructure of the NLHPC (ECM-02).”

"The funders had no role in study design, data collection and analysis, decision to publish, or preparation of the manuscript."

Please state what role the funders took in the study. If the funders had no role, please state: "The funders had no role in study design, data collection and analysis, decision to publish, or preparation of the manuscript."

We have added the statement “The funders had no role in study design, data collection and analysis, decision to publish, or preparation of the manuscript” in our cover letter.” Thanks for including this in the online submission.

If this statement is not correct you must amend it as needed.

Please include this amended Role of Funder statement in your cover letter; we will change the online submission form on your behalf.

Thanks so much for the clarification. Please modify our funding including the new grant and the following statement: "The funders had no role in study design, data collection and analysis, decision to publish, or preparation of the manuscript." As provided in the previous paragraph

3. PLOS requires an ORCID iD for the corresponding author in Editorial Manager on papers submitted after December 6th, 2016. Please ensure that you have an ORCID iD and that it is validated in Editorial Manager. To do this, go to ‘Update my Information’ (in the upper left-hand corner of the main menu), and click on the Fetch/Validate link next to the ORCID field. This will take you to the ORCID site and allow you to create a new iD or authenticate a pre-existing iD in Editorial Manager.

We confirm that the corresponding author’s ORCID iD (0000-0002-5073-7751) has been added and validated in Editorial Manager as instructed.

4. Please include captions for your Supporting Information files at the end of your manuscript, and update any in-text citations to match accordingly. Please see our Supporting Information guidelines for more information: http://journals.plos.org/plosone/s/supporting-information.

We have included it according to the guidelines.

5. We are unable to open your Supporting Information file supp. file 1 - network.cys and Supp. file2 - scripts.zip. Please kindly revise as necessary and re-upload.

To make this information available, we have repackaged the files and uploaded them to GitHub (https://github.com/JMALab/stress-ready) along with the code for this manuscript. Please note that the .cys file is provided exclusively in our GitHub repository due to system limitations regarding the number of characters in file names. Additionally, we have replaced the Supp. File 2 scripts with a notebook (S1 File) that can be opened using Jupyter or Google Colab.

[Note: HTML markup is below. Please do not edit.]

Reviewers' comments:

Reviewer's Responses to Questions

Comments to the Author

1. Is the manuscript technically sound, and do the data support the conclusions?

The manuscript must describe a technically sound piece of scientific research with data that supports the conclusions. Experiments must have been conducted rigorously, with appropriate controls, replication, and sample sizes. The conclusions must be drawn appropriately based on the data presented.

Reviewer #1: Partly

Reviewer #2: Yes

2. Has the statistical analysis been performed appropriately and rigorously?

Reviewer #1: Yes

Reviewer #2: I Don't Know

3. Have the authors made all data underlying the findings in their manuscript fully available?

The PLOS Data policy requires authors to make all data underlying the findings described in their manuscript fully available without restriction, with rare exception (please refer to the Data Availability Statement in the manuscript PDF file). The data should be provided as part of the manuscript or its supporting information, or deposited to a public repository. For example, in addition to summary statistics, the data points behind means, medians and variance measures should be available. If there are restrictions on publicly sharing data—e.g. participant privacy or use of data from a third party—those must be specified.

Reviewer #1: Yes

Reviewer #2: Yes

4. Is the manuscript presented in an intelligible fashion and written in standard English?

PLOS ONE does not copyedit accepted manuscripts, so the language in submitted articles must be clear, correct, and unambiguous. Any typographical or grammatical errors should be corrected at revision, so please note any specific errors here.

Reviewer #1: Yes

Reviewer #2: Yes

5. Review Comments to the Author

Please use the space provided to explain your answers to the questions above. You may also include additional comments for the author, including concerns about dual publication, research ethics, or publication ethics. (Please upload your review as an attachment if it exceeds 20,000 characters)

Reviewer #1: The manuscript by Contreras et al present a bioinformatics comparison of gene regulation in two close Solanum species using public data from a previous paper of their own. I like the style of the paper, straight to the point, but still I have comments for the authors:

Thanks to the reviewer for the positive comments.

L127 Please add FASTQC version

We have specified it in the manuscript (FastQC V.0.11.9).

L129 Please add GCA assembly codes for both genomes

We have specified it in the manuscript (GenBank accession ID GCA_000188115 for *S. lycopersicum* and GCA_001406875 for *S. pennellii*).

L129-131 Please rephraser and consider adding equation, in its current form it is not clear what's being done

We have rephrased the text and added the equation to describe the methodology more explicitly.

L133 Doesn't it make more sense to fit instead of assign?

We have not used the word "fit," but rather "assignment," because it is an algorithm with a rule-based decision tree structure. For this reason, the text was changed to clearly reflect this difference. Thank you very much for noticing that.

L135 Why were normalized expression values not computed by DEseq or kallisto, for instance in TPMs, why is the current approach used instead? Please justify. Also, are these the same values in Y-axis of Fig4B and Fig4C?

Thanks for this comment. Our approach was guided by the study by Eshel et al, in which stress ready states were also assessed (<https://doi.org/10.1111/nph.18411>). For this reason, we replicated the normalization method they proposed, which involves potential variations in gene lengths. Since this normalization could influence the classification or assignment of expression models, we also performed an alternative analysis in which we did not normalize by gene length, but only used DESeq2 normalization. This resulted in the same gene assignment; however, there were little variations in log2 fold change and adjusted and unadjusted p-values. Therefore, we have included the new DESeq2 outputs as supplementary material, and this has been mentioned in the manuscript (Line 136, page 7 and Line 236 page 10). Additionally, we have described the values for Y axis in Figure 4B and C.

L138 Please provide source code/or a URL to code for the decision tree and explan also how FC is computed

We have clarified in the text that the fold change (FC) values were derived using DESeq2. The tree-based algorithm (provided in Supplementary File 2: decision_tree_and_plots.ipynb) has now been referenced in the main text.

L165 Please add GOATOOLS version

We have specified it in the manuscript (GOATOOLS V1.4.12)

L173 Please add PLANTTFDB version and URL it possible

We have specified it in the manuscript (PlantTFDB V.5.0 - https://planttfdb.gao-lab.org/)

L174 Please explain the source of TSS or correct if you mean gene start; also explain whether raw or masked genomic sequences were used to scan motifs

We thank the reviewer for raising this point. The text has been modified to clarify that we used gene start coordinates. Also, raw genomic sequences were determined for motif detection to preserve original genomic context.

L198 The authors should compare their obtained total orthologues to other estimates in the literature or databases to give the reader a sense of how accurate their simple approach is; in particular, as they reckon, the reciprocal blast way is highly sensitive to paralogues and multiple copies. Same problem would apply to assigning Araport gene ids, which I don't get why it was required (sorry if I missed it).

We acknowledge the importance of comparing our results to external references. To address this, we compared the orthologs obtained using OrthoFinder with those obtained from other methods such as reciprocal BLAST. OrthoFinder yielded a smaller number of orthologs (orthogroups), many of which showed complex many-to-many relationships, likely due to the presence of paralogs. While this approach may underestimate the total number of orthologs compared to databases or other methods, it provides a more conservative and reliable framework for downstream expression analysis, where one-to-one orthologs are preferable to avoid ambiguity. We have clarified this point in the main text (Line 116 page 6, Line 220 page 10 and Line 478 page 20).

Regarding the BLAST comparison with Arabidopsis, this was used exclusively to assign gene symbols to tomato genes for ease of interpretation. This step was not used for ortholog detection, but only for annotation purposes. We will ensure this is clearly stated in the manuscript to avoid confusion (Line 118 page 6).

L211 Please help us understand these numbers: is this caused by the different number of genes in both species? Shouldn't both tests produce similar numbers of DEGs? Can this be explained by different variances across replicates?

Thank you for your thoughtful question. The difference in the number of DEGs between *S. lycopersicum* and *S. pennellii* is not due to the total number of genes annotated in each species, which are comparable. Instead, the variation in DEG counts likely reflects intrinsic biological differences in how each species responds transcriptionally to drought stress. This is consistent with the findings reported by Egea et al. 2018 (https://doi.org/10.1038/s41598-018-21187-2), who observed distinct transcriptomic responses under similar conditions.

To rule out the possibility that differences in DEG counts were due to technical variance across replicates, we performed Spearman correlation analyses between biological replicates, following ENCODE project recommendations (<https://www.encodeproject.org/data-standards/encode4-bulk-rna/>). These analyses showed high intra-species correlations (above 0.9), especially within the same condition, indicating strong consistency between replicates. Therefore, we conclude that the observed differences in DEGs are not driven by replicate variability but rather by true biological variation between species. This point has now been clarified further in the main text (Line 230 page 10).

L248 "minimal or no change" does not match FC > 0. Please see L138

Thank you for pointing this out. The confusion likely arises because the “No response” category includes three different cases. In this group, we observed no significant **within-species** differential expression under drought. However, we further compared gene expression **between species** (*S. lycopersicum* vs *S. pennellii*) to assess baseline differences. This cross-species comparison sometimes yielded a positive or negative fold change (FC > 0 or FC < 0), or no difference at all (FC ≈ 0). Therefore, FC values in the “No response” group do not reflect within-species regulation under drought, but rather interspecies expression differences under control or shared conditions.

To address this potential ambiguity, we have revised the figure caption to explicitly clarify that the fold changes in the “No response” category are derived from cross-species comparisons, and not from stress-induced changes within each species.

L274 Did the authors compute enrichment for random gene groups (negative control). These should increase the confidence on this section and would help put in context L294.

Thank you for this clarification. We have incorporated the suggested revisions and reported the updated results in the main text to improve clarity and ensure alignment with the methodology. This new analysis is explained in lines Line 178 page 8 and Line 347 page 14, and it did not show significant enrichment in random gene lists.

L307 Why was it neccessary to verify it, how often the decision groups genes with different responsed? I apologize if I misunderstood this point.

We apologize for the confusion and thank you for raising this point. The rationale behind this verification step was to ensure that the binary classification applied by the decision-tree-like algorithm (i.e., DE vs. non-DE, positive vs. negative fold change) accurately captured the underlying gene expression dynamics across conditions and species.

Although the classification method was consistent with our analytical pipeline, we wanted to confirm that it did not group genes with dissimilar expression patterns into the same category due to technical artifacts or borderline statistical thresholds. While this misclassification was not frequent, we considered it important to validate the robustness of the classification. Therefore, we visually inspected the expression patterns of genes within each category (as shown in Figure 4B), which confirmed that the assigned models reflect distinct and biologically meaningful gene expression responses.

This clarification has been incorporated into the revised manuscript in Line 330 page 14 to make our reasoning more transparent.

L339 See L274, same idea here; how often random gene lists are enriched in motifs? Moreover, please let us know how many motifs from each family were scanned so that readers can gauge whether they were all equally likely to start with. Perhaps this could go in Fig5. Moreover, as motifs in PLANTFDB are mostly predictions, it is possible that some motifs are matched more that others due to differences in motif quality. This limitation should be discussed. I think this part of the work would greatly improve if the most significant motifs reported were confirmed by an ab initio approach, for instance based on the over-representation of DNA sequences, without prior knowledge of the motifs.

As requested, we assessed how often random gene lists show motif enrichment. Using Fisher’s exact test on three independent sets of randomly selected promoters, we found that some motifs appeared enriched in only one replicate per expression model (see S7 Table). This indicates that occasional enrichment can occur by chance, but that consistent enrichment across replicates is rare, reinforcing the specificity of our results.

To provide more context, we now include in Figure 5 the number of motifs scanned per TF family. While ERFs have a high number of known motifs in PlantTFDB, other families with a similar number of motifs did not show significant enrichment, suggesting that enrichment is not solely driven by motif abundance.

We also acknowledge potential biases in motif quality, as PlantTFDB contains predicted motifs of varying reliability. To address this, we applied statistical testing individually to each motif and report only significant cases (Supplementary Figure 5), minimizing the influence of motif variability.

Finally, we complemented our analysis with *de novo* motif discovery. Notably, ERF-like motifs were independently recovered in *S. pennellii* (S6 Table), supporting the robustness of our findings and the biological relevance of enriched motifs in species-specific drought adaptation.

L378 It would help to see shared motifs as cis-elements mapped along orthologous promoters side to side, so readers can evaluate the conservation

We have added it in the manuscript in Line 416 page 17 (S6 Fig).

L414 I find it hard to understand that the GRN are so different, is it because the number of shared cis-elements across orthologous is minimal? Perhaps this number should go into table 3 somehow. Please explain.

Thank you for this insightful comment. The divergence between the GRNs of *S. lycopersicum* and *S. pennellii* arises in part because orthologous genes do not always share the same cis-regulatory elements in their promoter regions. This difference in motif presence likely reflects evolutionary rewiring of transcriptional regulation between the two species.

To better illustrate this, we have updated Table 3 to include not only the number of nodes, transcription factor–encoding genes, and edges, but also a summary of shared vs. species-specific cis-elements detected in orthologous gene promoters prior to and after motif filtering. This addition clarifies that while the initial GRNs were relatively similar in structure, the application of motif filtering based on promoter content revealed substantial divergence in regulatory potential. Only a small subset of orthologous genes retained shared binding motifs, which explains the limited overlap in final regulatory edges.

These differences are now highlighted in both the revised table and main text to better support the conclusion regarding species-specific regulatory network architecture (Line 451 page 18).

Reviewer #2: MAJOR ISSUES:

1. Why was Solanum chilense not considered for this study?

Thank you for your question. We did not include *Solanum chilense* in our study because the only available RNA-seq dataset for this species (PRJDB15063) uses a drought treatment protocol that differs markedly from our own. In that dataset, irrigation was withheld for 7 days, partially resumed, and samples were collected on day 12. In contrast, the *S. pennellii* dataset we used (PRJNA800740) applies a continuous drought treatment for 11 days, with sampling on day 11.

Due to these differences in drought exposure duration, recovery, and sampling time, including *S. chilense* would have introduced variability that compromises comparability across species. Our objective was to analyze species under similar experimental conditions to ensure consistency in stress response interpretation. For this reason, we limited our analysis to datasets that shared a comparable drought-induction methodology.

This clarification has now been included in the revised Discussion section of the manuscript in Line 494 page 20.

2. There are many instances where first person tone has been used for writing. Please consider using the third person, this is more formal and acceptable.

Thank you for pointing this out. We have made the change in the text.

3. In many instances, there is no consistency in the use of terminology.

Thank you very much for this comment. We have revised and modified the instances that we considered inconsistent in the text.

4. In many instances, some sentences are long and complex, which might hinder readability. Consider breaking down complex sentences into simpler ones for better clarity.

We have made changes to the text by separating ideas and clarifying various points that could potentially lead to confusion.

5. The discussion of ERF transcription factors and their roles in stress responses is good. However, it might benefit from a more detailed explanation of how these factors mechanistically contribute to stress resilience.

We have added information about the ERF transcription factor family and discussed how, mechanistically, they could be driving the response observed in the third case of the stress-ready pattern (Line 548 page 22 and Line 560 page 23).

6. There is should a section for conclusion and future directions. Which is missing.

Although this section is not mandatory in PLOS ONE, we have included it to highlight future directions and how these results could be utilized going forward.

MINOR ISSUES:

1.Line 114: "BLASTp" should be defined for readers unfamiliar with it, perhaps as "Basic Local Alignment Search Tool for proteins (BLASTp)."

Thank you for pointing this out. We have made the change in the text.

2.Line 198-201: The numbers of unique genes are inconsistent. If 21,104 is the number of orthologous genes, the unique genes should differ. Double-check these figures for accuracy. Consider rephrasing for clarity: "Additionally, we identified 13,325 unique genes in S. lycopersicum and 21,104 in S. pennellii."

Apologies for the confusion — it was an error in the text and has now been corrected.

3.Line 202-203: The explanation for non-orthologous genes is clear but consider adding more detail on how paralogs might affect the analysis.

Thank you for pointing this out. We performed a comparison using Orthofinder, an alternative methodology for the identification of orthologs/paralogs, and we discussed in the text the benefits of other techniques that allow the identification of potential paralogs (within orthogroups). We also addressed how this impacts the analysis when determining different expression models.

4. Line 204-205: Specify the conditions of the "control" and "drought" treatments for clarity.

We have added details on how the control and drought conditions were carried out.

5. Line 206: Moreira et al. (2022) is not properly cited.

Apologies for the error. Now the study has been correctly cited.

6. Line 207-209: The sentence is complex; consider breaking it into two for clarity. For example, "PCA revealed distinct expression patterns in orthologs. Species differences influenced these patterns, consistent with clade distances, and by environmental conditions such as drought."

We have rephrased the text for clarification.

7. Line 210-214: The comparison of differentially expressed genes is clear but ensure that the methodology for determining differential expression is described elsewhere in the document. Consider adding a brief discussion on the biological significance of the findings, especially regarding the implications of differential gene expression in response to drought.

We have expanded the discussion of these drought-related implications in the revised manuscript (Line 502 and Line 517 on page 21).

8. Line 231-233: Consider briefly explaining why the decision-tree algorithm method was chosen and how it categorizes orthologs.

Thank you for your comment. To clarify, our approach does not use an established method (i.e. machine learning algorithm). Instead, we designed a custom rule-based framework that resembles a tree-like structure, relying on predefined "if–else" logic based on two variables: log₂ fold change and adjusted p-value. This method categorizes orthologous genes into specific expression response models by sequentially evaluating whether a gene is differentially expressed in each species and whether the direction of expression change is concordant or divergent.

We chose this approach for its transparency and biological interpretability. Such rule-based categorization allows direct control over classification criteria, which is critical when interpreting gene expression patterns across species. To avoid confusion, we have revised the main text to clearly indicate that our method is logic-based and implemented by us (Line 145 page 7).

9. Lines 266-273: The statement "S. pennellii appears to be preadapted to drought" is a strong conclusion. Consider discussing potential evolutionary mechanisms or historical environmental conditions that may have contributed to this preadaptation.

Thanks for pointing this. We have elaborated further on this topic, particularly regarding the evolutionary mechanisms that the ERF transcription factor family contributes to drought resistance (Line 562 page 23).

10. Lines 279-284: Consider rephrasing for clarity: "Orthologs with increased expression under drought in both species were enriched for genes involved in seed dormancy, water deprivation, response to abscisic acid (ABA), and oxidative stress."

We have rephrased the text for clarification.

11.Lines 333-339: Consider rephrasing for clarity: "Using the FIMO tool, we scanned transcription factor binding motifs (TFBMs) in the promoters of genes associated with each expression model in both species.

We have rephrased the text for clarification.

12.Line 432: "pathways pathway" is a repetition. It should be corrected to "pathways."

We have deleted the repeated word.

13. Line 454: The sentence structure is awkward: "Notably, nearly one-third of drought-responsive orthologs were associated with a stress-ready state, representing 20% of all orthologs analyzed." Consider rephrasing for clarity: "Notably, nearly one-third of drought-responsive orthologs, representing 20% of all orthologs analyzed, were associated with a stress-ready state."

Thanks so much for the suggestion. We have rephrased the text.

14. Line 460: The phrase "providing weaker evidence for pre-adaptation in that context r (6)" contains a typographical error with the letter "r." It should be removed.

Sorry for the typo. We have removed the r.

15. Line 471: The phrase "but S. pennellii appears to adopt a more energy-efficient strategy during stress" could be more concise: "suggesting that S. pennellii adopts a more energy-efficient strategy during stress."

We have rephrased the text for clarification.

16. Line 481: The sentence is split awkwardly: "ERF proteins are multifunctional regulators known to enhance resilience to various stressors, including drought, salinity, extreme temperatures, and heavy metals, and are involved in processes such as root hair development, leaf senescence, and proline synthesis for osmoprotection (42)." Consider breaking it into two sentences for clarity.

We have rephrased the text for clarification.

7. PLOS authors have the option to publish the peer review history of their article (https://journals.plos.org/plosone/s/editorial-and-peer-review-process#loc-peer-review-history). If published, this will include your full peer review and any attached files. Do you want your identity to be public for this peer review? YES

7a. If you answered "Yes" above, that you would like your identity to be public. Vishnutej Ellur

6. PLOS authors have the option to publish the peer review history of their article (what does this mean?). If published, this will include your full peer review and any attached files.

If you choose “no”, your identity will remain anonymous but your review may still be made public.

Do you want your identity to be public for this peer review? For information about this choice, including consent withdrawal, please see our Privacy Policy.

Reviewer #1: No

Reviewer #2: Yes: Vishnutej Ellur
